# Research Inefficiency in Degenerative Cervical Myelopathy: Findings of a Systematic Review on Research Activity Over the Past 20 Years

**DOI:** 10.1177/2192568219847439

**Published:** 2019-06-12

**Authors:** Oliver D. Mowforth, Benjamin M. Davies, Samuel Goh, Cormac P. O’Neill, Mark R. N. Kotter

**Affiliations:** 1 Division of Neurosurgery, Department of Clinical Neurosciences, University of Cambridge, United Kingdom; 2 Wellcome Trust and MRC Cambridge Stem Cell Institute, Anne McLaren Laboratory, University of Cambridge, United Kingdom; * Joint first authors.

**Keywords:** cervical cord, cervical vertebrae, spondylosis, spinal osteophytosis, spinal cord diseases, chronic disease

## Abstract

**Study Design:**

Scoping review.

**Objective:**

To describe activity, themes and trends in degenerative cervical myelopathy (DCM) research over the past 20 years with a view to considering DCM research inefficiency.

**Methods:**

A systematic review of MEDLINE and Embase for “Cervical” AND ”Myelopathy” was conducted following PRISMA guidelines. Full-text papers in English, exclusively studying DCM, published between January 1, 1995 and December 31, 2015 were considered eligible. Country of origin, number of papers published, number of patients studied, research theme, and year of publication were assessed. Comparison was made between developed and developing countries.

**Results:**

A total of 1485 papers and 4 117 051 patients were included. Japan published more papers (450) than any other country while the United States studied the greatest number of patients (3 674 737). Over 99.4% of papers and 78.6% of patients were from developed countries. The number of papers (*r* = 0.96, *P* < .001) and patients (*r* = 0.83 *P* < .001) studied each year increased significantly overall and for both developed (*r* = 0.93, *P* < .001; *r* = 0.81, *P* < .001) and developing countries (*r* = 0.90, *P* < .001; *r* = 0.87, *P* < .001). Surgery was the most prevalent theme (58.3% papers; 55.7% patients) for developed and developing countries. Research from developing countries showed greater thematic variability.

**Conclusions:**

DCM research activity is increasing internationally, with surgery remaining the focus. Research output has predominantly been from developed countries; however, the rate of growth for developed and developing countries is comparable.

## Introduction

Degenerative cervical myelopathy (DCM) is a disabling syndrome of symptomatic spinal cord compression secondary to degenerative changes in the cervical spine.^1,2^ DCM is common,^2–4^ with an estimated prevalence of up to 5% in the population older than 40 years,^1^ which is expected to increase as global populations age.^2^ DCM patients experience a wide range of disabling symptoms.^1^ Limb pain, weakness, stiffness, and numbness are prevalent. Neck stiffness and neck pain are commonly reported, as are loss of dexterity, bladder and bowel dysfunction, and gait problems.^5^ Ultimately the disease is progressive^6^ and can, in very severe cases, result in paralysis.^7^

Surgical decompression, independent of surgical approach, is currently the only evidence-based treatment that has been shown to halt disease progression, but existing neurological damage is often permanent.^8,9^ Even with current gold standard surgical care, most patients retain lifelong disabilities.^9^ A recent study demonstrated that DCM severely affects quality of life; Short Form–36 health survey (SF-36) scores in DCM patients were lower than most chronic diseases including, cancer, hypertension, chronic lung disease, diabetes, and depression.^10^

One implication is a large and growing economic burden from the lifelong loss of productivity and the lifelong health and social care that DCM patients often require.^7^ A large and increasing clinical need exists to optimize care and reduce suffering, which must promptly be addressed by health care research.

Substantial international investment is made in research by governments and charities, but often with poor efficiency and failure to meet patient needs. This results in research waste^11^ with both economic and human consequences,^11^ serving to decelerate the speed of progress through mechanisms including failure to consolidate currently available evidence. Whilst some inefficiency is unavoidable, much cannot be justified. In their seminal paper, Chalmers et al^12^ identified a number of factors contributing to research wastage, including research duplication and failure to establish research priorities. Reviewing what is already known and setting research priorities before conducting further primary research is key to reducing inefficacy.^12^

The objective of this review is to provide a systematic overview of global DCM research from the past 20 years, identifying the research type, location, themes and trends including areas of potential inefficiency and comparison between developed and developing countries.

## Methods

A systematic review was conducted in accordance with the PRISMA guidelines.^13^ A search of Embase and MEDLINE for [“Cervical”] AND [”Myelopathy”] for papers published from January 1, 1995 to December 31, 2015 was performed for all studies considering cervical myelopathy secondary to chronic compression of the spinal cord.^14,15^ Animal studies, case reports, letters, editorials, reviews, technical notes, commentaries, proposals and corrections were excluded.

Titles and abstracts were screened for relevance, and subsequently full-text papers were screened for eligibility according to the following criteria:

- Primary clinical trial
- DCM is the primary condition being addressed in the paper
- English, full text

Papers were screened by 3 authors [SG, CO, ODM] and data was extracted independently by 2 authors [SG, CPO] using a piloted proforma. Discrepancies were settled by discussion and mutual agreement.

Following screening the following information was recorded for each paper:

- Country of corresponding author
- Year of publication
- Research theme
- Number of patients
- Whether a surgical or a nonsurgical cohort was studied
- Whether investigating the surgery itself was the primary objective of the research study

Research themes were initially developed using included studies from our previous systematic reviews.^14,15^ These themes were then piloted and iteratively refined in a random subset of 200 papers shortlisted for this review until a mutually exclusive and usable taxonomy (as mutually agreed by all authors) had been developed. A hierarchy was used such that each paper was categorized into the single theme which the authors felt best described the overall focus of the paper. The following 8 themes were used for categorization:

1. *Functional outcome* measures and their validation, such as the Japanese Orthopaedic Association Myelopathy Evaluation Questionnaire (JOAMEQ)
2. *Imaging* and its role in diagnosis or follow-up
3. *Molecular* and genetic investigations
4. *Prognostication* based on patient demographics and baseline characteristics
5. *Electrophysiological* investigations
6. *Surgical* technique, approach or strategy as the focus of the paper
7. *Epidemiological* studies
8. *Other* papers not covered by the above themes

The CIA World Factbook definitions were used to divide countries into developed or developing categories.^16^

Statistical analysis was used to detect trends in research themes across time. Correlations were performed using Spearman’s rank correlation coefficient, using SPSS Version 25 (IBM Corporation, Armonk, NY). Significance was set at *P* < .05. We report mean ± standard deviation unless otherwise specified.

## Results

**Figure 1.**
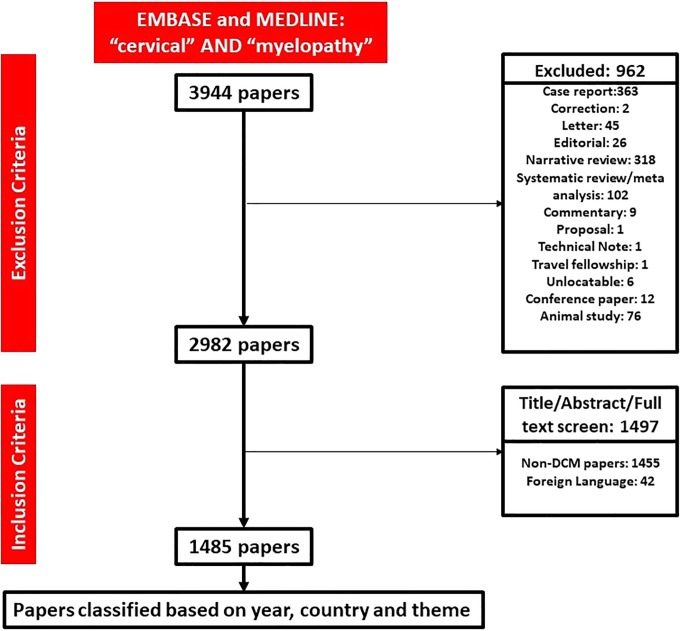
PRISMA (Preferred Reporting Items for Systematic Reviews and Meta-Analyses) flow diagram of search strategy.

The search strategy returned 3944 papers, of which 962 were excluded. Following title, abstract, and full-text screening, a total of 1485 full-text papers were included, assessing 4 117 051 patients (Figure 1). A total of 79% (1174) of papers studied a surgical cohort, with 45% (675) of total papers having surgical technique as the dependent variable.

**Figure 2.**
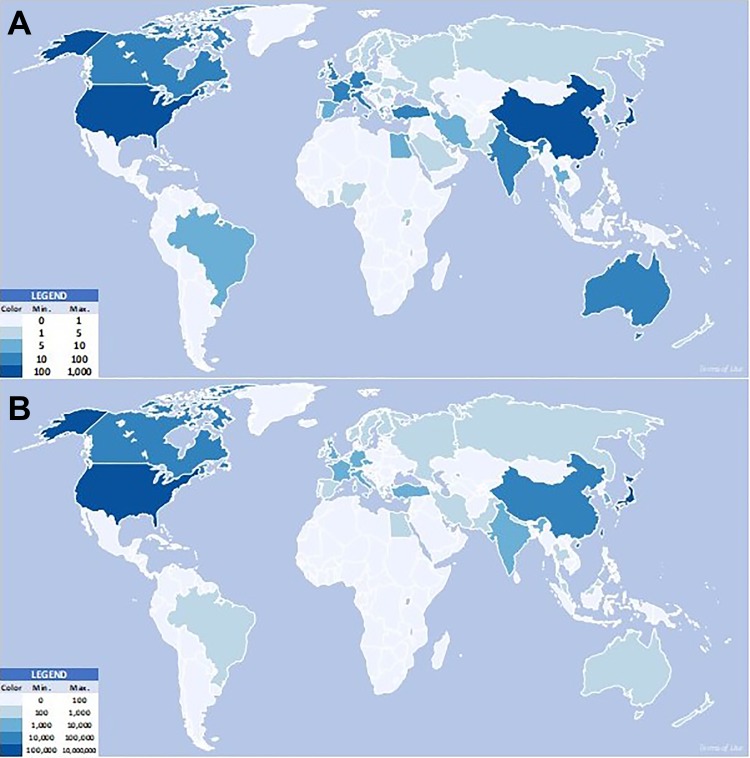
World heatmaps showing degenerative cervical myelopathy (DCM) research output by country between 1995 and 2015 in terms of (A) number of papers and (B) number of patients. The higher the output the darker the shade of blue in which the country is represented. Japan and the United States were the greatest producers of DCM research.

Between 1995 and 2015, DCM research was conducted in 53 countries (Figure 2). Japan was the country with the highest output of DCM research in terms of number of papers published, with 30.3% of total DCM papers over the 20-year period (Table 1); United States (19.5%) and China (12.0%) were the second and third most research-active countries in terms of papers published. However, United States studied the greatest number (89.2%) of DCM patients. Japan (5.6%) and Taiwan (3.6%) were the second and third most research-active in terms of number of DCM patients studied (Table 2).

**Table 1.** Top 10 Countries by Degenerative Cervical Myelopathy Research Paper Output Between 1995 and 2015.^a^

Country	Papers, n (%)	*r*	*P*
Japan	450 (30.3)	0.82	.001
United States	290 (19.5)	0.86	.001
China	178 (12.0)	0.88	.001
South Korea	71 (4.8)	0.87	.001
Canada	57 (3.8)	0.83	.001
India	57 (3.8)	0.77	.001
Germany	49 (3.3)	0.48	.027
United Kingdom	41 (2.7)	0.53	.013
Italy	34 (2.3)	0.33	.146
Czech Republic	28 (1.9)	0.47	.031
Other	230 (15.6)	0.92	.001

^a^ Spearman’s correlation coefficients were calculated to identify trends in growth of paper output by country (*r*) for which significance testing was conducted and *P* values are reported.

**Table 2.** Top 5 Countries by Number of Degenerative Cervical Myelopathy Research Patients Between 1995 and 2015.^a^

Country	Patients n (%)	*r*	*P*
United States	3 674 737 (89.2)	0.74	.001
Japan	230 176 (5.6)	0.92	.001
Taiwan	148 206 (3.6)	0.85	.001
China	16 597 (0.4)	0.80	.001
Canada	14 670 (0.4)	0.82	.001
Other	32 665 (0.8)	0.83	.001

^a^ Spearman’s correlation coefficients (*r*) were calculated to identify trends in the number of patients on which data was published each year by each country. Significance testing was conducted and *P* values are reported.

**Figure 3.**
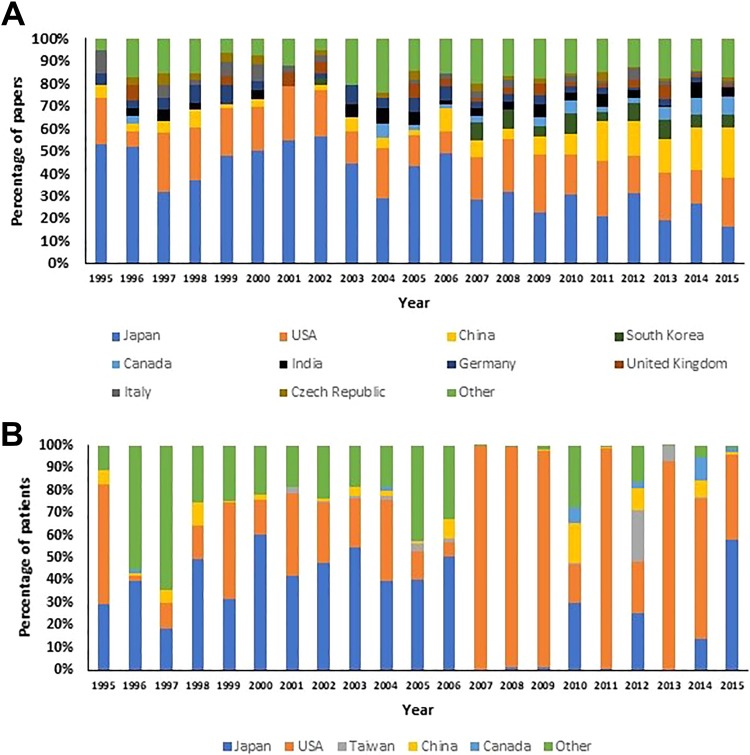
(A) Top 10 countries in terms of number of degenerative cervical myelopathy (DCM) research papers published. Japan was dominant in the first decade, publishing 32% to 56% of total papers each year. In the second decade, Japan*’*s output was frequently 30% or less of total international output. (B) Top 5 countries by number of patients studied. Japan and the United States were consistently the top 2 countries in terms of number of DCM patients studied each year. The relative dominance of the United States in 2007-2009 was due to a peak in number of patients studied in US studies.

The number of countries participating in DCM research became more diverse over the study period. In 1995, 19 DCM studies were published from 6 countries, while in 2015, 189 DCM studies were published from 30 countries (Figure 3), a statistically significant increase, *r* = 0.96, *P* < .001.

The number of patients studied per year increased over the study period from 1577 patients in 1995 to 330 935 in 2015, a statistically significant increase, *r* = 0.83 *P* < .001. However, this overall trend was accentuated by several US national database studies with very high patient numbers between 2006 and 2015.^17–19^

While the absolute number of papers from Japan increased from 10 in 1995 to 31 in 2015, the percentage of DCM papers from Japan fell from 52.6% in 1995 to 16.4% in 2015 (Figure 3A). The percentage of papers from the United States was relatively constant between 1995 (21.1%) and 2015 (21.7%). The absolute number of papers from China increased from 1 in 1995 to 41 in 2015, an increase in percentage of total papers from 5.3% in 1995 to 22.2% in 2015.

In the first decade, Japan was dominant in publishing data on the greatest number of patients (Figure 3B). In the second decade, papers with very high patient numbers from the United States led to the United States becoming the country studying the greatest number of patients per year and the greatest number in total over the whole study period.

A total of 28 developed and 25 developing countries published DCM research between 1995 and 2015, using CIA World Factbook definitions.^16^ Developed countries published 1167 (78.6%) studies on a total 4 092 626 (99.4%) patients compared with 318 (21.4%) studies on a total of 24 425 (0.6%) patients from developing countries.

**Figure 4.**
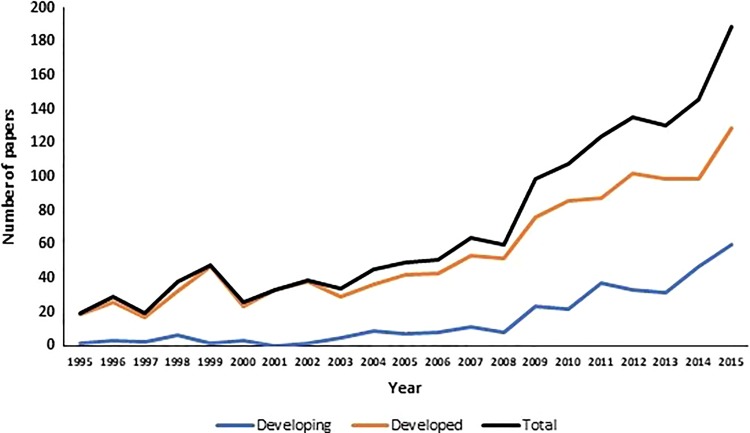
Number of papers published increased significantly overall and for both developed and developing countries between 1995 and 2015.

Between 1995 and 2015, developed countries showed a significant increase both in number of papers published, *r* = 0.93 *P* < .001 and in number of patients studied, *r* = 0.81, *P* < .001. In the same time period, developing countries showed comparable significant increases both in number of papers published, *r* = 0.90, *P* < .001 and in number of patients studied, *r* = 0.87, *P* < .001 (Figure 4). In context, over the same 1995-2015 period, the total number of papers published per year indexed in PubMed increased significantly with a coefficient of *r* = 0.99, *P* < .001.

In terms of number of papers, surgery was by far the most prevalent theme with a total of 866 papers, making up 58.3% of total DCM papers (Table 3), followed by imaging, which was the subject of 17.5% of total papers.

**Table 3.** Degenerative Cervical Myelopathy Research by Theme.^a^

Theme	Number of papers (%)	*r*	*P*	Number of patients (%)	*r*	*P*
Surgery	866 (58.3)	−0.05	.819	2 294 089 (55.7)	0.12	.610
Imaging	260 (17.5)	0.02	.947	18 564 (0.4)	**−0.76**	**.001**
Prognostication	115 (7.8)	**0.45**	**.042**	170 741 (4.1)	0.20	.372
Electrophysiology	91 (6.1)	**−0.58**	**.006**	6712 (0.2)	**−0.59**	**.005**
Functional Outcome	61 (4.1)	**0.53**	**.015**	8777 (0.2)	0.07	.749
Other	45 (3.0)	−0.36	.109	146 309 (3.6)	−0.27	.242
Molecular	27 (1.8)	0.33	.139	2457 (0.1)	**0.62**	**.003**
Epidemiology	20 (1.4)	0.33	.149	1 469 402 (35.7)	0.42	.061

^a^Surgery and imaging were the most prevalent themes, together accounting for over 75% of papers published between 1995 and 2015. Spearman’s correlation coefficients (*r*) were calculated to assess trends in percentages of papers and patients for each theme between 1995 and 2015, for which significance testing was conducted and *P* values are reported. Significant findings are in boldface.

Surgery was the research theme for which the greatest number of patients were studied, commanding a total of 2 294 089 (55.7%) patients (Table 3). While few in number (n = 20), the large patient numbers in epidemiological studies led to this theme being second most prevalent in terms of patient numbers with a total of 1 469 402 (35.7%) patients.

**Figure 5.**
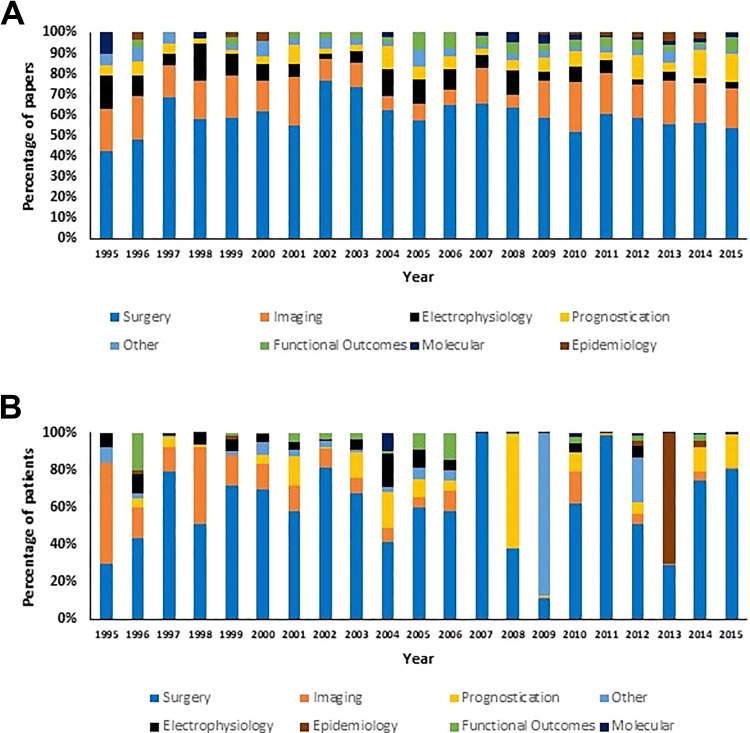
Trends in degenerative cervical myelopathy (DCM) research themes between 1995 and 2015. (A) In terms of number of papers, surgery was the most prevalent DCM research theme every year between 1995 and 2015. (B) In terms of number of patients, surgery remained the most prevalent research theme, while epidemiology was the second most prevalent theme.

In terms of number of papers, surgery was the most prevalent theme in every year of study between 1995 and 2015 (Figure 5A), consistently representing 40% to 75% of papers each year. Imaging was consistently the second most prevalent research theme with 10% to 20% of papers published on this theme each year.

Between 1995 and 2015, there was a small but significant increase in the percentages of prognostication papers, *r* = 0.45, *P* = .042 and functional outcome papers, *r* = 0.53, *P* = .015. In addition, there was a small but significant decrease in the percentage of electrophysiology papers, *r* = −0.58, *P* = .006.

Surgery remained the most prevalent DCM research theme when data was analyzed in terms of patient numbers (Figure 5B). However, there was more variability in the percentage of patients in surgically themed papers between years compared with the percentage of papers (Figure 5A and B). Epidemiology was the second most prevalent DCM research theme in terms of patient numbers, although the majority of these patients were represented in a small number of large studies, particularly in 2013.

Over the study period 1995-2015, there was a significant decrease in the percentage of patients in imaging studies, *r* = −0.76, *P* < .001, and electrophysiology studies, *r* = −0.59, *P* = .005. There was a significant increase in number of patients included in molecular studies, *r* = 0.62, *P* = .003.

**Figure 6.**
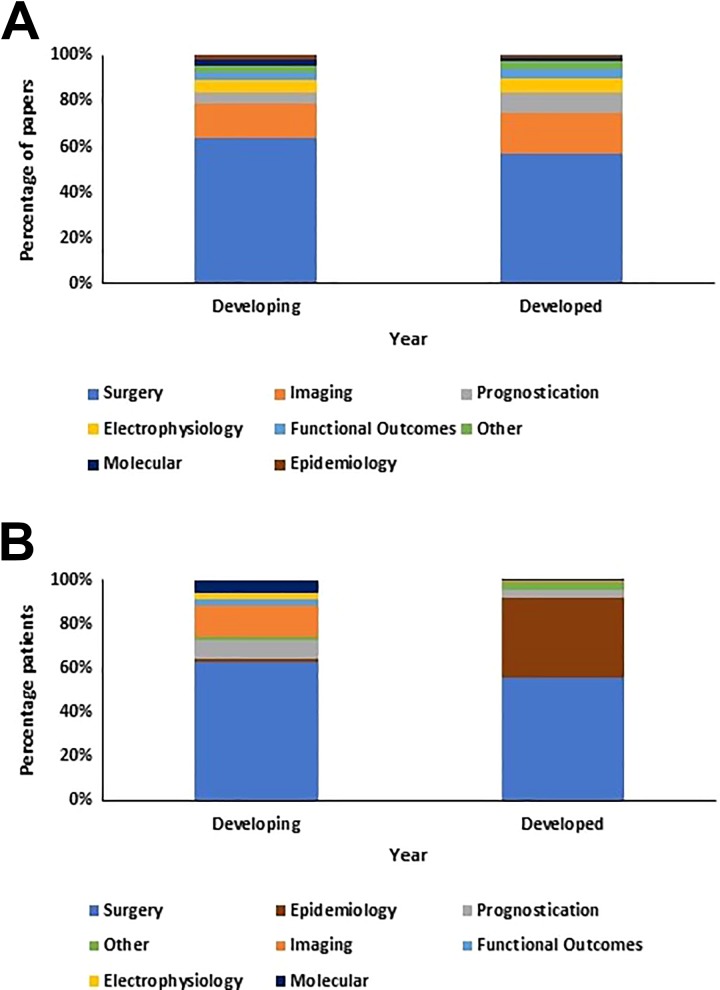
Research themes for developed and developing countries. (A) The percentage of papers published on each degenerative cervical myelopathy (DCM) research theme was similar for developed and developing countries. (B) Percentage distributions of patients between each DCM research theme differed for developed and developing countries, with developing countries showing greater diversity in patient distribution to themes.

The percentage of papers of each research theme were similar for developed and developing countries (Figure 6A). The highest percentage of papers were published on a surgical theme for both developed (56.8%) and developing (63.8%) countries. The second highest percentage of papers were published on imaging for developed countries (18.2%) and developing countries (15.1%).

Surgical themed papers included the largest percentage of DCM patients in both developed (55.7%) and developing countries (62.7%). However, the percentage distributions for number of patients in each theme were not similar for developed and developing countries (Figure 6B).

In developed countries, 35.9% of DCM patients were included in epidemiological papers, compared to 1.6% of patients in developing countries. In contrast, 14.1% of patients were included in imaging papers in developing countries, compared with 0.4% in developed countries. Whilst the surgical theme was world-wide predominant, there was more diversity in study themes for developing countries with higher percentages of patients included in papers studying prognostication, functional outcomes, electrophysiology, and molecular themes. In contrast, the majority of nonsurgical DCM patients in developed countries were included in epidemiological papers.

## Discussion

DCM research is international and increasing year-on-year, both in terms of number of papers published and number of patients studied. This includes both developed and developing countries, where growth is comparable. The majority of research is evaluating patients undergoing surgery, and in particular the surgery itself. Although significantly lower in number, growing areas of research interest include prognostication, development of functional outcomes, and molecular studies.

### Is Surgery Being Overresearched?

DCM treatment is predominantly managed by surgeons, with decompressive surgery the only evidence-based treatment.^8,9^ It has been shown to halt disease progression and offer meaningful, albeit generally incomplete, recovery. Consequently, a focus on surgical research might be expected and we identified that 79% of papers studied patients undergoing surgery, with 45% of papers evaluating the benefit of a surgical technique. This is in keeping with recent review of the top 100 most-cited spinal surgery papers,^20^ which included 3 studies of DCM, all of which concerned surgical technique.

This particular focus is likely to represent an example of research inefficiency for a number of reasons. First, not all patients undergo surgery. It is currently estimated that most DCM patients never gain a diagnosis^1,21,22^ and not all patients are managed with surgery.^22–24^ Second, fundamentally important aspects of DCM, such as the natural history of disease and its exact etiology remain unknown.^25^ Third, the synthesized evidence of surgical research has not found additional benefit for one surgical approach over another.^23^ Fourth, despite a recognized need, little research has aimed at finding strategies to improve recovery.^26,27^ Finally, the majority of DCM research consists of low evidence design.^14^

In an attempt to provide high-level evidence on the significance of surgical technique, the ongoing Cervical Spondylotic Myelopathy Surgical (CSM-S) Trial (NCT02076113) is due to report and may offer some closure here.^28^ Nevertheless, there are important surgical questions that remain to be answered. For example, we have previously demonstrated that most studies have focused on patients undergoing surgery for the first time and the role of repeat surgery remains relatively unexplored.^14^ Additionally the evidence base defining the timing, especially for acute central cord syndrome without instability, remains weak.^23^

In summary, while ongoing surgical research is required, its present direction likely contains inefficiencies. Global energy and enthusiasm could be better harnessed to address knowledge gaps.

### The Past and the Future

We found significant increases in absolute output in all 8 DCM research themes. While surgery remained predominant, there was relative growth for prognostication, functional outcome and molecular research studies. Conversely, there has been a relative decrease in electrophysiology studies.

These emerging themes, from our perspective, align with important and unaddressed issues. The natural evolution of DCM is unknown and currently unpredictable^25^; in some individuals, symptoms remain mild over extended periods of time while in others, disease progression is accelerated.^23,29^ This uncertainty creates a number of clinical challenges, none more so than the timing of surgery^23^ and detection of myelopathy from asymptomatic spinal cord compression.^30^

Most existing DCM assessments rely on qualitative grading^14^ with few examples of quantitative assessments, and these are therefore poorly placed to answer current research questions.^31^ Moreover, current diagnostic tools have been unable to represent spinal cord damage. This has undoubtedly led the move toward new assessment techniques, including diffusion tensor imaging^32^ and gait analysis.^33^ Beyond tracking differing disease profiles, there is increasing interest in the biological basis of DCM, such as genetics.^34^ A better understanding of the disease process will contribute to new treatment development.

The reasons for declining interest in electrophysiology is unclear; as a quantitative assessment tool it could be an improvement on current outcomes assessments.^14,31^ Moreover, it has been shown to be of value in detecting subclinical myelopathy.^35^ The potential is likely undermined with declining access and patient interest; in the United Kingdom, almost every hospital has a magnetic resonance imaging scanner but very few have neurophysiology services and in our experience, patients tolerate but would prefer not to undergo the procedure.

As depicted in Figure 6B, the percentage of patients in epidemiological papers is much smaller for developing countries compared with developed countries. This likely reflects the lack of national data banks in many developing countries, which is a key area of development for the future.

### Research From Developing Countries

The Global Forum for Health Research estimated that total public funding for health research in developing countries was US$2.5 billion per year, representing just 3% of total global funding for health research.^36^ In contrast, at the turn of the millennium, low and middle-income countries were estimated to represent 85% of the world’s population, 92% of global disease burden but just 10% of global funding for health research.^37^ Despite this, the United Nations Educational, Scientific and Cultural Organisation estimates that 27% of researchers work in developing countries.^38^

Indeed, DCM is a global problem,^9^ which is increasingly common with global aging populations^39^ and our finding of active and growing research participation in developing countries is reassuring, and aligns with the World Health Organization objective that “all nations should be producers and users of research.”^40^

The involvement of developing countries can also offer novel insights. An important example of this within neurosurgery is the BEST TRIP trial,^41^ which conducted a randomized trial of intracranial pressure monitoring for traumatic brain injury. For DCM, where the prevalence of DCM subtypes differs with ethnicity,^2^ this may be essential.

### Increasing Research Efficiency

Health research inefficiency is ubiquitous.^11^ However, other fields have acknowledged and taken steps to counteract this through research priority setting partnerships, which are taking place internationally^42^ and recognized to improve efficiency.^12^ The James Lind Alliance in the United Kingdom, Global Evidence Mapping in Australia, and the Deep Inclusion Method/Choosing All Together in the United States, are examples of multistakeholder partnerships involving patients and the public that have enjoyed success.^42^ Such partnerships have already shown promise in diabetes,^43^ head and neck cancer,^44^ psoriasis,^45^ and kidney cancer^46^ among many other diseases.

In DCM, in the context of our findings and the many significant unmet needs, such a process is an important next step for the field. Partnerships involve patients, families, carers, and health professionals from multiple disciplines and can elicit and prioritize research questions. Based on our findings, this needs to be a global process, in order to support the clear, ongoing, international efforts to advance care in DCM.

### Limitations

This review was designed to provide an overview of DCM research, and consequently extracted common data elements only. Inherently, this will have created some limitations in the interpretation of the data. For example, papers were attributed to address of the corresponding author, which therefore will not have accounted for multinational research. Moreover, the grouping themes were developed by the authors, based on a more focused systematic^14,15^ review; retrospective and prospective cohorts were not separated. Additionally, no attempt was made to distinguish datasets used for multiple purposes; it is not possible to rule out inclusion of individual patients in more than one paper and therefore the number of patients studied is likely to be lower than reported. This said, we feel that the findings overall reflect the literature, and given the significant number of studies included, serve as an accurate overview of the field in the past 20 years.

## Conclusion

DCM research is conducted globally and is increasing year on year. Surgery has been a major focus of research so far, which has overlooked many important knowledge gaps. A priority-setting partnership would improve efficiency and allow the global appetite for research to be better harnessed in delivering much needed advancement.
